# Domestication Process of the Goat Revealed by an Analysis of the Nearly Complete Mitochondrial Protein-Encoding Genes

**DOI:** 10.1371/journal.pone.0067775

**Published:** 2013-08-01

**Authors:** Koh Nomura, Takahiro Yonezawa, Shuhei Mano, Shigehisa Kawakami, Andrew M. Shedlock, Masami Hasegawa, Takashi Amano

**Affiliations:** 1 Faculty of Agriculture, Tokyo University of Agriculture, Kanagawa, Japan; 2 School of Life Sciences, Fudan University, Shanghai, China; 3 Institute of the Statistical Mathematics, Tokyo, Japan; 4 Gunma Safari World co. Ltd, Gunma, Japan; 5 College of Charleston Department of Biology and Medical University of South Carolina College of Graduate Studies, Charleston, South Carolina, United States of America; Institute of Molecular Genetics IMG-CNR, Italy

## Abstract

Goats (*Capra hircus*) are one of the oldest domesticated species, and they are kept all over the world as an essential resource for meat, milk, and fiber. Although recent archeological and molecular biological studies suggested that they originated in West Asia, their domestication processes such as the timing of population expansion and the dynamics of their selection pressures are little known. With the aim of addressing these issues, the nearly complete mitochondrial protein-encoding genes were determined from East, Southeast, and South Asian populations. Our coalescent time estimations suggest that the timing of their major population expansions was in the Late Pleistocene and significantly predates the beginning of their domestication in the Neolithic era (≈10,000 years ago). The ω (ratio of non-synonymous rate/synonymous substitution rate) for each lineage was also estimated. We found that the ω of the globally distributed haplogroup A which is inherited by more than 90% of goats examined, turned out to be extremely low, suggesting that they are under severe selection pressure probably due to their large population size. Conversely, the ω of the Asian-specific haplogroup B inherited by about 5% of goats was relatively high. Although recent molecular studies suggest that domestication of animals may tend to relax selective constraints, the opposite pattern observed in our goat mitochondrial genome data indicates the process of domestication is more complex than may be presently appreciated and cannot be explained only by a simple relaxation model.

## Introduction

The goats (*Capra hircus*) are one of the oldest domesticated animals, and based on archaeological evidence are thought to have been domesticated initially in the Fertile Crescent (≈10,000 years ago) [1]. A recent molecular study by Naderi et al. [2] suggested that goats were domesticated from bezoars (*C. aegagrus*) in West Asia. Afterward, goats spread globally and played an important role in the Neolithic agricultural revolution and advance of human civilization. Nowadays, goats are distributed on all continents excluding Antarctica, and are also found on many peripheral and remote islands. About 840 million goats are kept in the world spanning humid tropical rain forest regions, dry, hot desert regions, and cold, hypoxic high altitude regions [3], and provide essential sources of meat, milk, and fiber.

In the last decade, numerous detailed molecular phylogeographic studies of goats have been carried out to clarify their origin of domestication and their transportation routes [2], [4]–[10] mainly based on mitochondrial D-loop sequences. These extensive studies revealed that there are six major haplogroups in the mitochondrial lineages of goats, namely haplogroup A, B, C, D, F, and G. According to Naderi et al. [9], haplogroup A is the most frequent haplogroup and more than 90% of goats inherit this haplogroup, and are largely distributed throughout the Old World. In addition, goats in the New World (South and Central America) all belong to haplogroup A [10]. In contrast, the other haplogroups show regional distributions [9]. The distribution areas of haplogroup B (5.92% of all goats [9]) are mainly in East and Southeast Asia. Interestingly, this haplogroup is dominant in Southeast Asia in contrast to haplogroup A being dominant in other regions. Chen et al. [8] suggested a secondary origin of domestication in China, and haplogroup B arose in this region. From the distribution area of the bezoars and from the results of the molecular phylogeographic study by Naderi et al. [2], Chen et al [8]'s hypothesis seems unlikely because the bezoar is not distributed in East Asia [11], and haplogroup B can be observed in the bezoar from West Asia [2]. According to Naderi et al. [9], haplogroup B can be divided into the sub-haplogroup B1 and sub-haplogroup B2. Haplogroup C (1.44%) is mainly distributed in the European region. Haplogroup D (0.54%) is mainly in South and Central Asia. Haplogroup F (0.12%) is in Sicily, and Haplogroup G (1.11%) is in West Asia. Naderi et al. [2], [9] showed that the phylogenetic relationships among these haplogroups are (F,(C,(B,(G,(D,A))))) based on the mitochondrial D-loop, and studies based on the D-loop are essentially all concordant with these relationships.

Despite this phylogenetic concordance, the coalescent times of these haplogroups remain controversial. The estimated time of the MRCA (most recent common ancestor) of goats (based on the split of haplogroup C from others in the simplified sampling scheme of early studies) were from 201,380 to 597,806 years ago [4], [6], [8]. These estimates were mainly based on 3^rd^ codon positions of the mitochondrial *cytochrome b* gene assuming a divergence time between goat and sheep at 5∼7 Ma [12], [13]. The timing of the population expansion of goats is also unclear. Luikart et al. [4] estimated the timing of population expansion of haplogroup B (≈2,130 years ago) and haplogroup C (≈6,110 years ago) assuming that the expansion of haplogroup A occurred ≈10,000 years ago (approximate time for initial domestication). However, Fang and Anderson [14] suggested that population expansion of the Asiatic pigs occurred around 275,000 years ago and about 190,000 years ago for European pigs which drastically predates the beginning of pig domestication (about 9,000 years ago) based on analysis of the D-loop. To avoid circular arguments, the assumed date for calibration should be independent from the evidence for domestication events. In this case, the biological or geological events at the inter-species level should be used as calibration points. Horai et al. [15] demonstrated that the use of mitochondrial genomes can provide accurate high-resolution estimates of intra-species coalescent times as compared to only D-loop sequence. Thus, in this study we used nearly complete mitochondrial protein coding genes to estimate the coalescent times of the major haplogroups of goats.

The second point that requires clarification is the difference in selection pressure among different lineages which can also be revealed accurately by complete mitochondrial genome analysis. Recently, Björnerfeldt et al. [16] and Wang et al. [17] indicated a higher ω (non-synonymous to synonymous rate ratio) in domesticated animals compared with their wild progenitors. They interpreted this to indicate a relaxation of selective constraints during domestication. However, little is known about the difference between selective constraints at early versus late phases of the domestication process and goats provide a valuable opportunity for investigating this issue. As mentioned above: (1) goats have a long domestication history; (2) goats are distributed in variable environments all over the world and haplogroup A covers all areas, whereas the other haplogroups are distributed regionally; and (3) multiple maternal lineages were involved in the domestication process.

The aim of this study is to analyze a nearly complete set of mitochondrial protein encoding genes to reveal: 1) the molecular evolutionary circumstances prior to goat domestication (e.g., the coalescent times of major lineages and the timing of population expansion); 2) the process at the onset of domestication (e.g., the bottleneck and selection profile in wild progenitors); and 3) the posterior profile of domestication (e.g., the differential selection pressures before, during and after domestication).

## Results

### Phylogenetic tree

**Figure 1:**
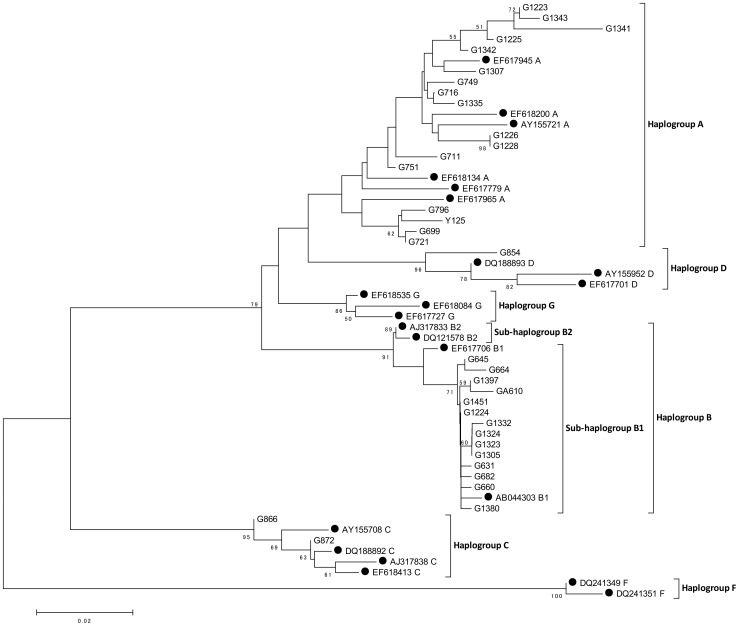
NJ tree based on the D-loop sequences of goats: Kimura's 2 parameter model [35] with the gamma distribution (α = 0.22) was used in estimating genetic distances. Branch lengths are proportional to the number of nucleotide substitutions. The markhor was used as an outgroup. Nodal numbers indicate bootstrap probabilities (10,000 replications).

The NJ tree based on the mitochondrial D-loop is shown in Figure 1. Our samples were classified into four haplogroups, namely A, B, C and D. None of our samples came from the haplogroup F or haplogroup G. Monophyly of each haplogroup was supported with relatively high bootstrap values (>80%) except for haplogroup A (46%).

Haplogroup A consists of the breeds from Mongolia (Mongolian indigenous goat), Japan (Japanese Saanen), Korea (Korean indigenous goat), Indonesia (Etawa), Bangladesh (Black Bengal) as well as two wild individuals of the bezoar (*C. aegagrus*). Haplogroup B consists of the breeds from Indonesia (Kambing Katjang, Etawa), Bangladesh (Black Bengal), and the Philippines (Philippine indigenous goat). All of them are belonging to the sub-haplogroup B1. Haplogroup C consists of the breed from Mongolia (Mongolian indigenous goat). Haplogroup D also consists of the breeds from Mongolia (Mongolian indigenous goat) (Table 1). In previous studies, samples from Southeast Asia were quite limited (e.g., [9]) especially from island nations such as Indonesia and the Philippines. The ML tree based on the nearly complete mitochondrial protein-encoding genes (Figure S1) also shows a largely concordant branching pattern with the D-loop data. In the study of Naderi et al. [9] on the basis of D-loop data, bootstrap support values for haplogroup A were low (53%), and substantially higher values (94%) were obtained in the present analysis. The phylogenetic relationships among haplogroups are harmonious with previous studies [2], [4], [6]–[9]. Although our samples do not include haplogroup F or G, the bootstrap values of the internal nodes are relatively high (94% for haplogroup A+D; 99% for haplogroup A+D+B).

**Table 1:** Summary of sample information for the present study.

haplogroup	breed	smaller data1	individual numbers	undetermined regions2						
				protein encoding regions						D-loop
				ND2	CO1	CO2	ATP8	ND3	ND5	HV1
**A**	Mongolian native	**<$>\raster="rg1"<$>**	**G699^a^**							
		**<$>\raster="rg1"<$>**	**G711**							
			**G716**						•	
			**G721^a^**							
		**<$>\raster="rg1"<$>**	**G749**							
			**G778**		•					•
		**<$>\raster="rg1"<$>**	**G796**							
	Japanese saanen	**<$>\raster="rg1"<$>**	**G1226**							
		**<$>\raster="rg1"<$>**	**G1228**							
	Korean native	**<$>\raster="rg1"<$>**	**Y125**							
	Indonesian Etawa		**G1307**		•					
	Bangladeshi Black Bengal	**<$>\raster="rg1"<$>**	**G1223**							
		**<$>\raster="rg1"<$>**	**G1225**							
		**<$>\raster="rg1"<$>**	**G1335**							
		**<$>\raster="rg1"<$>**	**G1341**							
		**<$>\raster="rg1"<$>**	**G1342**							
		**<$>\raster="rg1"<$>**	**G1343**							
**B**	Indonesian Kambing Katjang		**G631^b^**							
		**<$>\raster="rg1"<$>**	**G645**							
		**<$>\raster="rg1"<$>**	**G660**							
		**<$>\raster="rg1"<$>**	**G664**							
		**<$>\raster="rg1"<$>**	**G665^b^**							
		**<$>\raster="rg1"<$>**	**G682**							
	Indonesian Etawa	**<$>\raster="rg1"<$>**	**G1305**							
		**<$>\raster="rg1"<$>**	**G1323**							
		**<$>\raster="rg1"<$>**	**G1324**							
			**G1332**		•					
	Bangladeshi Black Bengal	**<$>\raster="rg1"<$>**	**G1224^c^**							
			**G1334^c^**							•
		**<$>\raster="rg1"<$>**	**GA609**					•		
			**GA610**						•	
	Philippine native		**G1380**			•				
		**<$>\raster="rg1"<$>**	**G1397**				•			
		**<$>\raster="rg1"<$>**	**G1451**				•			
**C**	Mongolian native	**<$>\raster="rg1"<$>**	**G866**							
		**<$>\raster="rg1"<$>**	**G872**							
**D**	Mongolian native	**<$>\raster="rg1"<$>**	**G854**							
		**<$>\raster="rg1"<$>**	**G725^d^**							•
		**<$>\raster="rg1"<$>**	**G739^d^**							•
		**<$>\raster="rg1"<$>**	**G751**							
**wild goat**	bezoar	**<$>\raster="rg1"<$>**	**G1239^e^**				•			
		**<$>\raster="rg1"<$>**	**G1247^e^**				•			
	markhor		**G1253**							

1 Samples that were used for the small data set indicated by <$>\raster="rg1"<$>.

2 Undetermined regions indicated by •.

a,b,c,d,e Identical haplotypes.

The sequences downloaded from NCBI (GU068049: haplogroup A; GU295658: haplogroup B) were analyzed for both small and large data sets.

Recently, Hassanin et al. [18] reported that several mitochondrial genome sequences of goats deposited in GenBank, including the reference sequence NC_005044 contain numts (pseudogenes of mitochondrial DNA transferred to the nuclear genome) and an unusually high percentage of sequence errors. These errors would tend to mislead inference of tree topologies. In order to evaluate the possible error caused by numts and other sources of sequence errors, we inferred the phylogenetic tree based on each of the 12 mitochondrial protein-coding genes separately. All 12 trees showed consistent topologies (data not shown), providing evidence that our data do not contain such spurious sequence regions.

### Time scale for the evolution and domestication of the goat

The estimated divergence times within the comprehensive evolutionary framework of the Cetartiodactyla are shown in Figure S2. The estimates from amino acid sequences and nucleotide sequences were mostly concordant. Remarkably, our estimates are 1.5 times older than those of Hassanin and Ronpiquet [19]. They assumed the Bovinae/Caprinae splitting at 18.5 Ma (mega annum). This is the younger limit for this splitting [20]. Accordingly, their estimates can be considered as minimal ages.

The Bovinae/Caprinae splitting was estimated to be 25.3±2.3 Ma (amino acid) and 27.6±1.0 Ma (nucleotide). The goat/sheep splitting was 14.7±2.1 Ma and 16.2±1.2 Ma. This is much older than the fossil calibrations used by Luikart et al. [4] (5∼7 Ma). This implies that if this younger fossil age is used as the calibration point, the divergence time will be grossly underestimated. The goat/Gobi ibex splitting was estimated to be 7.9±1.5 Ma and 7.9±1.0 Ma, and the goat/markhor splitting was 3.4±0.9 Ma and 3.4±0.8 Ma. As mentioned in the Materials and Methods, this age for the goat/markhor splitting was applied as the calibration point to estimate divergence times among extant goat haplogroups.

**Figure 2:**
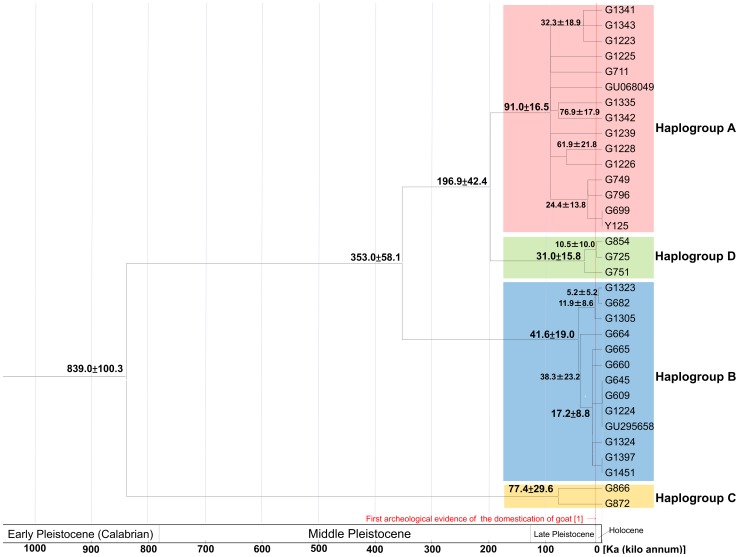
Coalescent time estimates for domestic goats based on 3^rd^ codon positions of the nearly complete mitochondrial protein-encoding genes of the smaller data set. The strict molecular clock method was applied (LRT: p-value = 0.233). Branch lengths are proportional to the estimated times. Nodal numbers indicate estimated divergence times with ± standard errors in Ka (kilo annum). Only the estimates of representative nodes are shown. The markhor was used as an outgroup and the goat/markhor split was assumed to be 3400 Ka (see main text).

The estimates are shown in Figure 2. The split between haplogroup C and the other haplogroups was 0.84±0.1 Ma, and the split between haplogroup B and haplogroup A + D was 0.35±0.08 Ma. These estimates are much older than those of previous studies [4], [6], [7].

We also estimated the times of MRCAs of each haplogroup. Since our samples widely cover each haplogroup (Figure 1), the MRCAs of our samples are expected to approximate real MRCAs of domestic populations in each haplogroup. The times of the MRCA of haplogroup A was estimated to be 90,950±16,460 years ago, the haplogroup B (sensu stricto sub-haplogroup B1) was 41,930±18,980 years ago, the haplogroup C was 77,350±29,570 years ago, and the haplogroup D was 32,300±18,910 years ago. Our sample mainly consists of haplogroup A and haplogroup B (sub-haplogroup B1). Both of them show star-like branching patterns (the asterisks in Figure 2) and age estimates of 90,950±16,460 years ago (haplogroup A) and 17,210±8,900 years ago (sub- haplogroup B1), respectively.

### ω (Non-synonymous rate/synonymous rate) ratios among lineages

Based on the ML tree topology from the nearly complete mitochondrial protein-encoding genes, we estimated the ω ratios for the inter-species branches (0.0564 for the smaller data, 0.0609 for the larger data), the deep branches (0.0448, 0.0478), and the shallow branches (0.1107, 0.1020). See Materials and Methods regarding the definitions for “smaller and larger data” and “deep and shallow branches”.

**Figure 3:**
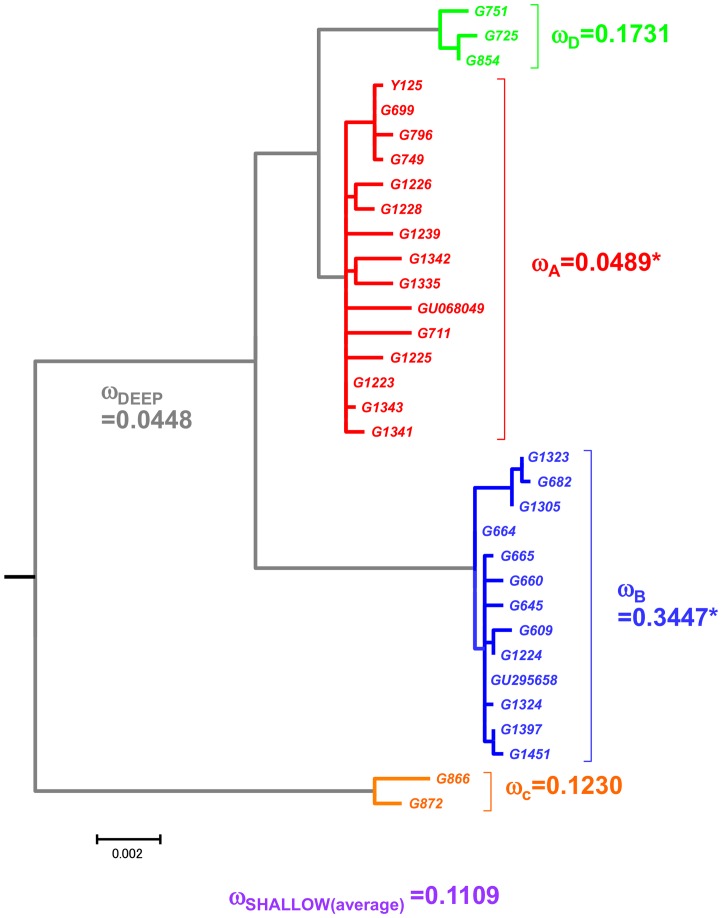
Differences of ω ratios among goat lineages based on the smaller data set of nearly complete mitochondrial protein encoding genes. The branch model analysis assuming different ω ratios in the shallow branches (a); and the branch model analysis assuming different ω ratios in the deep branches (b). Branch lengths are proportional to the numbers of codon substitutions.

Among shallow branches, the ω ratios were different for different haplogroups (Figure 3a and Figure S3a). The ω ratios of haplogroup A (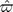 **_A_**) were 0.049 (smaller data set) and 0.053 (larger data set). Those of haplogroup B (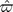 **_B_**) were (0.345, 0.387), those of haplogroup C (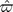 **_C_**) were (0.123, 0.123), and those of haplogroup D (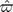 **_D_**) were (0.173, 0.259).

The ω ratios of the deep internal branches were also estimated. These branches were defined as follows: ω_a_ (common ancestral branch of haplogroup A), ω_b_ (common ancestral branch of haplogroup B), ω_c_ (common ancestral branch of haplogroup C), ω_d_ (common ancestral branch of haplogroup D), ω_x_ (common ancestral branch of haplogroup A+D), and ω_y_ (common ancestral branch of haplogroup A+D+B). The estimates of these ω ratios are shown in Figure 3b (based on the smaller data set) and Figure S3b (based on the larger data set), respectively. The ω ratios for the deep branches were as follows: 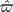
_a_ (0.2206, 0.1451), 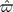
_b_ (0.1483, 0.1395), 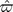
_c_ (0.0246, 0.0360), 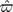
_d_ (0.0356, 0.0360), 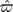
_x_ (0.0001, 0.0001), 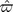
_y_ (0.0001, 0.0001). The average ω ratio of these six deep branches was 0.0448∼0.0478, as mentioned above.

## Discussion

### Differences of selection pressure

The ω ratios of the deep branches (0.0448∼0.0478) were almost the same as the ratios of inter-species branches (0.0564∼0.0609). This implies that the slightly deleterious mutations [21] were mostly swept out from these ancestral lineages [22]. On the other hand, there was a statistically significant difference between the deep branches (0.0448∼0.0478) and the shallow branches (0.1020∼0.1107) based on a likelihood ratio test (LRT); p-values were 0.032 (smaller data set) and 0.041 (larger data set) (Table 2: 3ω model vs. 2ω model). This suggests that most of the slightly deleterious mutations are still retained in the extant populations.

**Table 2:** The model comparisons for the different ω ratios in the shallow branches.

model		lnL^1^	#p^2^	AIC^3^		LRT^4^ *
Small data set						
1ω model		−15205.52	126	30663.03		
2ω model	inter-species ≠. intra-species	−15205.12	127	30664.23	0.37	vs. 1ω model
3ω model	inter-species≠ deep ≠ shallow	−15202.82	128	30661.64	**0.032**	vs. 2ω model
4ω1 model	inter-species≠ deep ≠ shallow (A≠B = C = D)	−15198.96	129	30655.91$	**0.005**	vs. 3ω model
4ω2 model	inter-species≠ deep ≠ shallow (B≠A = C = D)	−15199.23	129	30656.47	**0.007**	vs. 3ω model
4ω3 model	inter-species≠ deep ≠ shallow (A = B≠C = D)	−15202.70	129	30663.39	0.619	vs. 3ω model
5ω model	inter-species≠ deep ≠ shallow (A≠B≠C = D)	−15198.24	130	30656.48	0.232	vs. 4ω1 model
6ω model	inter-species≠ deep ≠ shallow (A≠B≠C≠D)	−15198.20	131	30658.40	0.775	vs. 5ω model
Large data set						
1ω model		−16099.94	138	32475.88		
2ω model	inter-species ≠. intra-species	−16099.50	139	32477.00	0.347	vs. 1ω model
3ω model	inter-species≠ deep ≠ shallow	−16097.02	140	32474.05	**0.026**	vs. 2ω model
4ω1 model	inter-species≠ deep ≠ shallow (A≠B = C = D)	−16091.78	141	32812.48$	**0.001**	vs. 3ω model
4ω2 model	inter-species≠ deep ≠ shallow (B≠A = C = D)	−16092.75	141	32467.49	**0.003**	vs. 3ω model
4ω3 model	inter-species≠ deep ≠ shallow (A = B≠C = D)	−16096.62	141	32475.23	0.367	vs. 3ω model
5ω model	inter-species≠ deep ≠ shallow (A≠B≠C = D)	−16091.16	142	32466.33	0.268	vs. 4ω1 model
6ω model	inter-species≠ deep ≠ shallow (A≠B≠C≠D)	−16090.92	143	32467.85	0.489	vs. 5ω model

1 lnL (log-likelihood score), ^2^#p (numbers of parameter), ^3^AIC (Akaike Information Criterion), ^4^LRT (p-value of the likelihood ratio test).

$ the minimal AIC (best models).

* p-value <5%.

There were also significant differences of ω ratios within the shallow and deep branches, respectively. Concerning the shallow branches, the 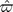 **_A_** (0.049∼0.053) is substantially smaller, and 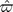 **_B_** (0.345∼0.387) is substantially higher than those of the other ratios. To evaluate the difference of ω in each haplogroup, the LRT was applied comparing the variable levels of heterogeneity of ω among lineages. The results are summarized in Table 2. There was no significant difference between ω**_C_** and ω**_D_**. Therefore, we assumed these two haplogroups to be homogenous and estimated the average of ω**_C_** and ω**_D_** (ω**_C+D_**)**.** Moreover the differences between ω**_C +D_** and ω**_B_**, and ω**_C+D_** and ω**_A_** were also not significant (5ω model vs. 4ω1 model, see table 2; 5ω model vs. 4ω2 model data not shown). However, the difference between ω**_A_** and ω**_B_** was significant (Table 2). Insignificant difference between ω**_C+D_** and ω**_B_**, or ω**_C+D_** and ω**_A_** is probably due to the small sample size in our data set (2 sequences for haplogroup C, and 3 for haplogroup D). This analysis suggests that high selection pressure has continued to operate on haplogroup A.

The results of the McDonald and Kreitman's Test [23] are shown in Table 3. When haplogroup A and other haplogroups were compared, the differences of the synonymous and non-synonymous substitutions among inter- or intra-haplogroups were not significantly different. However, when the other haplogroups were compared (e.g., haplogroups B vs. haplogroups C, haplogroups B vs. haplogroups D, and haplogroups C vs. haplogroups D), the differences were significant. In the latter cases, the relative non-synonymous substitution numbers were higher in the intra-haplogroup than in the inter-haplogroup. In contrast, the relative non-synonymous substitution numbers among haplogroup A are almost the same with that of the inter-haplogroup. This also supports the hypothesis that most of the non-synonymous substitutions have already been swept out from haplogroup A.

**Table 3:** The results of the McDonald and Kreitoman's test [23] based on the complete mitochondrial protein encoding genes.

larger data#										
	Total number of codons analyzed	type of substitution	fixed	polymorphic	Neutrality Index	α value	Fisher's exact test.	G value	Williams' correction	Yates' correction
haplogroup A vs. haplogroup B	1961	synonymous	18	28	1.768	−0.768	0.539	0.787	0.757	0.324
		non-synonymous	4	11				<0.375>	<0.384>	<0.569>
haplogroup A vs. haplogroup C	2396	synonymous	42	42	1.500	−0.500	0.740	0.360	0.343	0.070
		non-synonymous	4	6				<0.548>	<0.558>	<0.792>
haplogroup A vs. haplogroup D	2396	synonymous	11	38	1.013	−0.013	1.000	0.000	0.000	0.169
		non-synonymous	2	7				<0.988>	<0.989>	<0.681>
haplogroup B vs. haplogroup C	1962	synonymous	40	13	4.923	−3.925	0.018*	6.190	5.903	4.701
		non-synonymous	5	8				<0.0129*>	<0.015*>	<0.030*>
haplogroup B vs. haplogroup D	1962	synonymous	23	8	7.667	−6.667	0.011*	7.526	7.143	5.642
		non-synonymous	3	8				<0.00608**>	<0.00753**>	<0.01754*>
haplogroup C vs. haplogroup D	3561	synonymous	75	12	7.813	−6.813	0.008**	7.480	6.742	5.560
		non-synonymous	4	5				<0.00624**>	<0.00942**>	<0.01837*>

smaller data$										
	Total number of codons analyzed	type of substitution	fixed	polymorphic	Neutrality Index	α value	Fisher's exact test.	G value	Williams' correction	Yates' correction
haplogroup A vs. haplogroup B	3395	synonymous	26	51	0.874	0.126	0.794	0.063	0.061	0.000
		non-synonymous	7	12				<0.8012>	<0.80421>	<0.98538>
haplogroup A vs. haplogroup C	3395	synonymous	64	49	3.048	−2.048	0.183	2.658	2.529	1.668
		non-synonymous	3	7				<0.10305>	<0.11178>	<0.19654>
haplogroup A vs. haplogroup D	3395	synonymous	14	47	1.043	−0.043	1.000	0.002	0.002	0.136
		non-synonymous	2	7				<0.96114>	<0.96274>	<0.71184>
haplogroup B vs. haplogroup C	3395	synonymous	72	16	5.625	−4.625	0.001882**	9.925	9.523	8.272
		non-synonymous	8	10				<0.00163**>	<0.00203**>	<0.00403**>
haplogroup B vs. haplogroup D	3395	synonymous	36	14	3.673	−2.673	0.038549*	5.089	4.914	3.873
		non-synonymous	7	10				<0.02408*>	<0.02665*>	<0.04908*>
haplogroup C vs. haplogroup D	3395	synonymous	74	12	8.222	−7.222	0.015468*	6.326	5.528	4.387
		non-synonymous	3	4				<0.01190*>	<0.01871*>	<0.03621*>

# The numbers of the sequence in each haplogroup are as follows: haplogroup A (18 sequences), haplogroup B (17 sequences), haplogroup C (2 sequences), haplogroup D (3 sequences).

$ The numbers of the sequence in each haplogroup are as follows: haplogroup A (15 sequences), haplogroup B (13 sequences), haplogroup C (2 sequences), haplogroup D (3 sequences).

The statistical significance.

* p-value <5%.

** p-value <1%.

Concerning the deep branches, 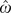
_b_ (0.1395∼0.1483) is significantly higher than others, and 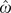
_y_ (0.0001) is significantly smaller than others (Table 4: 4ω2 model vs. 3ω model, 4ω6 vs. 3ω model). The extremely small ω ratios can be expected in this case due to their association with deep ancestral branches. Although 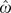
_a_ (0.1451∼0.2206) is relatively large, it was not significantly larger than that of other branches, probably due to its short branch length (Table 4: 4ω1 model vs. 3ω model).

**Table 4:** The model comparisons for the different ω ratios in the deep branches.

model		lnL^1^	#p^2^	AIC^3^	LRT^4^ *	
Small data set						
3ω model	inter-species≠ deep ≠ shallow	−15202.82	128	30661.64		
4ω1 model	inter-species≠ deep ≠ shallow (a≠b = c = d = x = y)	−15202.13	129	30662.25	0.239	vs 3ω model
4ω2 model	inter-species≠ deep ≠ shallow (b≠a = c = d = x = y)	−15199.09	129	30656.17	**0.006** *	vs 3ω model
4ω3 model	inter-species≠ deep ≠ shallow (c≠a = b = d = x = y)	−15202.27	129	30662.55	0.296	vs 3ω model
4ω4 model	inter-species≠ deep ≠ shallow (d≠a = b = c = x = y)	−15202.79	129	30663.58	0.810	vs 3ω model
4ω5 model	inter-species≠ deep ≠ shallow (x≠a = b = c = d = y)	−15202.19	129	30662.38	0.261	vs 3ω model
4ω6 model	inter-species≠ deep ≠ shallow (y≠a = b = c = d = x)	−15200.09	129	30658.19	**0.020** *	vs 3ω model
5ω model	inter-species≠ deep ≠ shallow (b≠y≠a = c = d = x)	−15197.69	130	30655.38$	**0.028** *	vs 4ω6 model
Large data set						
3ω model	inter-species≠ deep ≠ shallow	−16097.02	140	32474.05		
4ω1 model	inter-species≠ deep ≠ shallow (a≠b = c = d = x = y)	−16096.63	141	32475.26	0.376	vs 3ω model
4ω2 model	inter-species≠ deep ≠ shallow (b≠a = c = d = x = y)	−16093.99	141	32469.97	**0.014** *	vs 3ω model
4ω3 model	inter-species≠ deep ≠ shallow (c≠a = b = d = x = y)	−16096.85	141	32475.71	0.560	vs 3ω model
4ω4 model	inter-species≠ deep ≠ shallow (d≠a = b = c = x = y)	−16096.98	141	32475.96	0.764	vs 3ω model
4ω5 model	inter-species≠ deep ≠ shallow (x≠a = b = c = d = y)	−16096.35	141	32474.69	0.244	vs 3ω model
4ω6 model	inter-species≠ deep ≠ shallow (y≠a = b = c = d = x)	−16094.20	141	32470.39	**0.017** *	vs 3ω model
5ω model	inter-species≠ deep ≠ shallow (b≠y≠a = c = d = x)	−16092.34	142	32468.67$	0.054	vs 4ω6 model

1 lnL (log-likelihood score), ^2^#p (numbers of parameter), ^3^AIC (Akaike Information Criterion), ^4^LRT (p-value of the likelihood ratio test).

$ the minimal AIC (best models).

* p-value <5%.

### Evolutionary history of domestic goat

**Figure 4:**
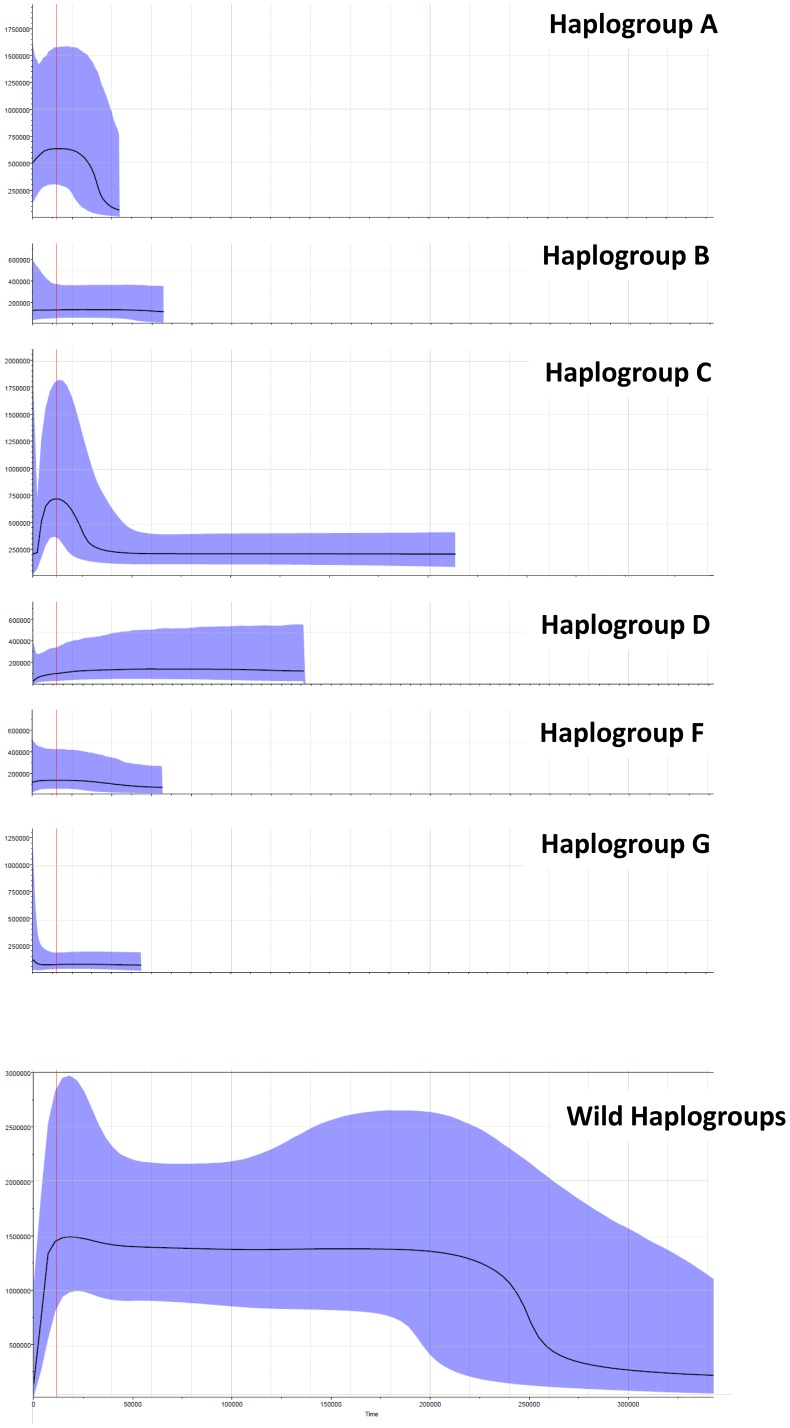
Bayesian Skyline Plot analysis of bezoar (*Capra aegagrus*) population size fluctuation. X axis indicates time scale (years before present), thin auxiliary lines indicate 20,000 year increments, bold auxiliary lines indicate 100,000 year increments. Y axis indicates effective population size multiplied by generation time. For the bezoar, females become sexually mature at 2–3 years [11]. Thin auxiliary lines indicate 500,000 increments, bold auxiliary lines indicate 1,000,000 increments.

Our estimated times for the MRCAs of each haplogroup (32,300∼90,950 years ago) and the times of nodes with star-like branching pattern (17,210 years ago and 90,950 years ago) substantially predate the beginning of goat domestication (about 10,000 years ago) [1]. Does this mean the population expansions of the wild progenitors of domestic goats occurred prior to the domestication events? Fang and Anderson [14] also reported a similar result for D-loop sequences of domestic pigs. Their mismatch distribution analysis suggested that the expansions of European and Asian domestic pig populations occurred nearly 190,000 and 275,000 years ago, respectively, and these estimated times extensively predate the domestication of the pig (9000 years ago) [24]. In this and Fang and Anderson's study [14], only domestic populations were used. Therefore, we re-analyzed D-loop sequences of the wild bezoars reported by Naderi et al. [2]. Although not significant in most cases, bezoars that are close to domestics (haplogroup A∼F, except haplotype D) show negative values for Tajima's D [25]. This implies recent weak population expansion events. Conversely, all bezoars that are not close to domestics (wild haplogroups) show positive values for Tajima's D (Table S1). The results of the Bayesian Skyline Plot analysis also indicate the population expansion events occurred prior to domestication (Figure 4). Haplogroups A and C show conspicuous, rapid expansions, and haplogroups B and G show slow expansions. In contrast to Tajiama's D, population size of haplogroup F has slowly declined.

To explain the population growth of the wild progenitors, two scenarios are possible. The first is Horwitz's incipient domestication [26]. Naderi et al. [2] suggested that “this evidence of a population growth suggests a phase of demographic control and protection of some populations of bezoars in the wild, before the isolation of true early domestic herds by humans” (p.17661–17662). The second is that the population growth was not caused by human activity. It is likely, however, that such a well established, expanded population frequently encountered ancient humans and some of them were involved in the domestication process.

The fluctuation of the population size estimated by the Bayesian Skyline Plot reveals that the wild population not involved in domestication experienced a rapid expansion around 250,000 years ago, and subsequently remained relatively constant in size for a long time. From around 10,000 years ago, the population size suddenly showed a rapid decrease. The timing of this decline is nearly concordant with the beginning of domestication [1]. It is possible that this was due to human activities such as hunting, destruction of suitable habitat for the wild population, and resource competition with domestic populations.

Since our time estimates suggest the MRCAs of each haplogroup extensively predated the beginning of domestication, the difference of the ω ratios of the “deep” branches do not appear to bear directly on the process of domestication (e.g., human-mediated transportation, artificial selection of breeds, etc.), but rather on prior historical events (e.g., natural selection on wild progenitors and isolation of the wild progenitor population at the beginning of domestication). The extremely small value of 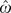
_x_ and 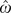
_y_ (0.0001) can be attributed to these being deep internal branches in which deleterious mutations have been largely swept out leaving mostly neutral fixed mutations.

The large value of 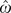
_b_ (0.1483) may be related to the isolation of wild progenitors at the beginning of domestication. In the case of haplogroup A, domestic goats seem to have been chosen widely from many lineages of wild progenitors ([2]'s Fig. 1). Similar patterns can be observed in haplogroups D and G. In contrast, domesticates in haplogroup B most likely were sampled more narrowly from a specific lineage of wild progenitors. We hypothesize that this biased isolation may have caused a severe bottleneck effect.

According to the nearly neutral hypothesis [21], [27], [28], negative selection is strict and slightly deleterious mutations are removed quickly in a large population, however, as population size gets small, slightly deleterious mutations behave as if neutral. Hence slightly deleterious mutations could be easily fixed in haplogroup B. Although similar biased isolation can be observed in haplogroup C ([2]'s Figs. 1 and 2), the ω ratio of this deep branch is smaller than those of other deep branches (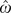
_c_ = 0.0246: smaller data). Naderi et al. suggested that domestic goats that inherit haplogroup C originated from a wild population in Eastern Turkey and that this population had relatively recently differentiated from a much larger Iranian (Zagros) population ([2]'s Fig. 2). Probably, after differentiation, most of the slightly deleterious mutations were swept out from the Turkish population for an unknown reason.

On the other hand, the difference of the ω ratios of the “shallow” branches seems directly related to the process of domestication. Here, we would like to focus attention on the small ω ratio of the haplogroup A. There are approximately 840 million goats in the world [3]. Naderi et al. [9] showed that more than 90% of them inherit the mitochondrial type represented by haplogroup A, and this is also the dominant type in most regions of the Old World. Moreover, Amills et al. [10] reported that all of the Central and South American goats inherit this type of mitochondria. In contrast, the population sizes of other haplogroups are much smaller and the distribution areas are limited. To take an example, sub-haplogroup B1 (second largest haplogroup) is inherited by 4.4% of the goats and their distribution areas are limited to East, Southeast, and South Asia. This can also be explained in the framework of the nearly neutral theory. As we mentioned above, the population size of haplogroup A is very large. In addition, it is the most dominant in the early stages of domestication (no less than 87% at the beginning of domestication: [2]). Accordingly, it can be implied that negative selection has been strict in such a large population and thus most of the slightly deleterious mutations would be removed by natural selection. Björnerfeldt et al. [16] and Wang et al. [17] reported higher ω ratios in domesticated animals than their wild progenitors, and suggested relaxation of selective constraints during domestication events. In addition, the reduction of the effective population size caused by a bottleneck at the beginning of the domestication process [29] and subsequent inbreeding during breeding-improvement seems to have contributed to the higher ω ratios observed in domesticated animals. In our study the samples of wild progenitors were limited (only 2 individuals), and therefore we could not evaluate the difference of the ω ratios between the domestic goat and the wild bezoars with statistical significance. The ω ratios of haplogroups B, C, and D (ω = 0.123∼0.387) were higher than those of the wild species [e.g., wild wolves (ω = 0.091: [16]), wild yaks (ω = 0.076: [17]), wild boars (ω = 0.105; Yonezawa and Hasegawa, unpublished data). This range of values is consistent with published values for other domestic animals like dogs (ω = 0.183 [16]), domestic yaks (ω = 0.231 [17]), and pigs (ω = 0.172; Yonezawa and Hasegawa, unpublished data). However, the ω ratio seen in haplogroup A was as low as the deep ancestral branches. This implies that most of the slightly deleterious mutations were already swept from the population, and that severe selective constraints have continued to operate in this haplogroup. The goats are also known as the “poor man's cow” [30] and are often not subject to highly developed industrialized agricultural practices. It is reasonable to expect that relatively strong selection pressures similar to those present in wild progenitors may have remained intact for domesticated goats that are subjected to extensive pastoralism such as being raised in semi-natural habitats or herded within completely natural free-range environments. The goat is also known to be one of the oldest domestic animals [1]. This implies that even though slightly deleterious mutations may have accumulated in the early phase of domestication, such mutations in a large and old population would be expected to be eventually eliminated from the population given a long enough time such as 10,000 years. Goats are also kept in widely variable environments spanning the humid tropical rain forest, the cold and hypoxic condition of the high altitude, extremely dry desert regions, and the remote isolated habitats of small islands resulting from human transportation. This broad diversity of conditions may have served to help keep selection pressure relatively intense in the goat population from historical periods up to the current state of goat breeding.

It is worth noting that our 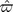 **_B_** (0.345, 0.387) is even higher than ratios reported for other domestic animals. In the present study, our data includes only sub-haplogroup B1 which is distributed widely in East, South, and Southeast Asia [9]. As such, the unique sub-haplogroup B2, which is observed only in goats kept in the China-Mongolian region and the wild bezoars in West Asia [2], [8], [9] are not included in our analysis. Chen et al. [8] pointed out that haplogroups B1 and B2 show star-like tree structures and that South Asia haplotypes are derived from the Eastern Asian haplotypes. Therefore, it is possible that the China-Mongolian region was a secondary domestication site that served as a “transportation” center in Asia. Since Southeast Asia is the only place where haplogroup A is not dominant, it is possible that there was a severe bottleneck in the transportation of goats from Western Asia to South Asia and Southeast Asian regions via China-Mongolia. Thus, the analysis of the mitochondrial genome data for haplogroup B2 should shed light on the enigmatic process of drift vs. selection operating during historical transportation of goats.

Regardless of these open questions, the extremely low ω ratio of haplogroup A and the extremely high ω ratio of haplogroup B add significant insight into the complex relationship between the population genetic structure of domestic animals and the importance of selection, breeding environment, demographic history, and the purpose of domestication in shaping their biological diversity.

## Conclusion

Our analyses of the nearly complete mitochondrial protein-encoding genes of the goat revealed that: (1) the timing of population expansion of goats occurred in the Late Pleistocene and extensively predates the beginning of goat domestication ∼10,000 years ago. This result is consistent with the population expansion of Asian and European pigs; (2) the ω ratio of the most dominant type represented by haplogroup A is extremely low which implies that most of the slightly deleterious mutations have been swept out of the goat population by selection. The apparently strong selective constrains in goats are probably due to the extremely large population size of haplogroup A present from the beginning of domestication, and also the highly variable and often extreme breeding environments of this animal; (3) conversely, the ω ratio of haplogroup B (both for shallow and deep branches) was extremely high. This suggests that the biased sampling of domestic goats from their wild progenitors in haplogroup B during the beginning of domestication, and subsequent transportation to South and Southeast Asian via China-Mongolia likely created extreme bottlenecks that facilitated fixation of slightly deleterious mutations in the lineage of this haplogroup.

## Materials and Methods

### Ethics Statement

All of the experimental works involving animals in this study followed the guidelines of the Animal Experimental Ethics Committee of the Tokyo University of Agriculture, Japan, and has been approved by them.

### Samples

Blood samples were collected from 450 goats within the original areas of the populations, for 9 breeds from 6 Asian countries, i.e., Japan, Korea, Mongolia, Indonesia, Philippines and Bangladesh (Table 1). The detailed information of the samples is described in Nomura et al. [31]. Blood samples of bezoar were collected from Gunma Safari World Co. Ltd, Japan, the markhor (*Capra falconeri*) was collected from the Yumemigasaki zoo, and the Gobi ibex (*Capra sibirica*) was provided by the Research Institute of Animal Husbandry, Mongolian State University of Agriculture.

### Experimental protocols

DNA was extracted from blood samples using a proteinase K digestion and a phenol-chloroform extraction [32]. To identify the mtDNA haplogroups, a 481 bp fragment of the first hyper-variable segment (HVI) of the control region was amplified for all samples using the primers D-HVI-CAP-FI and D-HVI-CAP-RI (Table S2), and sequenced. Subsequently, 40 goats that represent haplogroup A, B, C, and D (see below section “Haplogroup identification”) were selected, and each protein coding region was amplified and sequenced using 12 pairs of PCR primers (Table S2). PCR amplifications were conducted in 25 μl reaction volume with 1.5 mM MgCl_2_, 0.3μM of each primer, 200 μM of each dNTP, 1U TaKaRa Taq^TM^HS (TaKaRa BIO INC. Otsu, Japan) and 100 ng DNA. The PCR profile consisted of an initial denaturation step at 94°C for 5 min, 35 amplification cycles (denaturation at 94°C for 1 min, annealing at 52–66°C for 1 min and extension at 72°C for 2 min) and a final extension at 72°C for 10 min. PCR products were purified using the Wizard^®^ SV Gel and PCR Clean-Up System (Progema, Madison, WI, USA) and used for sequencing with the Big Dye^®^ Terminator v3.1 Kit (Applied Biosystems, Foster City, CA, USA) on an ABI prism 3100 Avant DNA analyzer. The sequences were aligned using the MEGA v. 4.0 program [33], against reference sequences for goat (GenGank accession number NC_005044), cattle (V00654) and sheep (AY858379). All newly determined sequence data were deposited in DDBJ (DNA Data Base of Japan: www.ddbj.nig.ac.jp/) and accession numbers were shown in Table S3.

### Haplogroup identification

Naderi et al. [9] analyzed the mitochondrial D-loop (hyper-variable region 1: HVR1) of 2430 domestic goats (1540 haplotypes) from all regions of the Old World and indicated that there are six major haplogroups (A, B, C, D, F, and G). They also characterized 22 reference sequences that define the 6 haplogroups. For the purpose of haplogroup identification, we inferred the phylogenetic tree based on the D-loop sequences of our samples together with these 22 reference sequences. The sequences were manually aligned and carefully checked by eye. The phylogenetic tree was inferred by the neighbor joining (NJ) method [34] with the K80+Г model [35], [36] using the MEGA v. 4.0 program [33]. The shape parameter (α) of the Г distribution was fixed at 0.22, as estimated using the BASEML program of PAML v. 4.2 [37]. The confidence values for internal branches were evaluated by the bootstrap method [38] with 10,000 replications.

### Alignment and inference of the phylogenetic tree based on the protein-encoding genes

The nucleotide sequences of protein-encoding genes on the H strand were manually aligned and carefully checked by eye. The following regions were excluded from the alignment: initiation and termination codons, and overlapping regions between *ATP6* and *ATP8*, *ND4* and *ND4L*, *ND5* and *ND6*. The mitochondrial genomes from two goats were downloaded from NCBI (GU068049, GU295658) and were aligned together with our original sequences. The markhor was included in this alignment as an outgroup.

Since we could not determine nucleotide sequences of all genes in several individuals, our full data set contains missing regions. Therefore, we made two sets of alignments, defining “the smaller data set” to have no gaps, and “the larger data set” to contain gaps and missing sequence regions. After concatenating all the data, identical sequences were excluded from the final alignment. The smaller data set consists of 34 individuals (33 goats and one markhor) with 10 genes (*ATP6, COX1, COX2, COX3, ND1, ND2, ND4L, ND4, ND5, cytochrome b*: 10,188 bp in total), and the larger data set consists of 40 individuals (39 goats and one markhor) with 12 genes (*ATP6, ATP8, COX1, COX2, COX3, ND1, ND2, ND3, ND4L, ND4, ND5, cytochrome b*: 10,683 bp in total). The list of the variant sites is shown in Table S3.

The phylogenetic tree was inferred by the maximum likelihood (ML) method [39] using the RAxML v. 7.0.3 program [40] with the GTR+I+Г_4_ model [36], [41], [42]. Taking into account the different tempo and mode of nucleotide substitutions, the three codon positions were analyzed separately. The branch lengths were also estimated independently. Gap sites were treated as missing data. To evaluate the confidence at nodes of the internal branches, we applied the rapid bootstrap method [40] with 1000 replications.

### Estimation of selection pressure and population expansion

To evaluate the selection pressure, the non-synonymous/synonymous rate ratio ω (dN/dS) was analyzed. The branch model that allows different levels of heterogeneity for the ω ratio among the lineages [43] was applied using the CODEML program in PAML v. 4.2 [37]. It is known that the amino acid substitution rate estimated by intra-species comparisons is much faster than those of inter-species comparisons, probably because slightly deleterious mutations are not completely swept out of populations within the smaller time scales of intra-species comparisons [22]. Moreover, the ω ratios of the shallow branches are usually much higher than those of the deep branches probably from the same reason [17], [44]. Therefore, we placed the branches into three distinct categories: (1) Shallow branches, which are defined as all terminal and internal branches descended from the MRCAs (most recent common ancestor) of each haplogroup; (2) Deep branches, which are defined as the internal branches between the MRCA of all goats and MRCAs of each of the haplogroups; and (3) Inter-species branches, which connect the markhor and the MRCA of all goats.

McDonald and Kreitman's Test [23] was also applied to evaluate differential selection pressure between intra- and inter-haplogroups using the DnaSP v. 4.2 program [45]. Tajima's D [25] was also estimated using the same program to detect recent population expansion events. Fluctuation of the ancestral population sizes were estimated by the Bayesian Skyline Plot method [46] using the BEAST program v. 1.7.4 [47] with the HKY+Г [36], [42] model under a strict clock. We newly estimated the mutation rate of D-loop for goats using our alignment for the “Haplogroup identification” with the BASEML program of PAML [37], assuming the MRCA of haplogroup A lived 91,000 years ago. This new rate was estimated at 2.73×10^−7^/site/year.

### Divergence times estimations

Previous studies [4], [6], [7] estimated the divergence times among the major goat haplogroups, assuming the goat/sheep split to be 5.0 to 7.0 Ma (mega annum) based on the ungulate fossil record [13]. However, the fossil record in general does not always point to the “real” divergence time because the first stratigraphic appearance of taxa in the fossil record may be subjected to sporadic sedimentary disruptions due to erosion or lack of sedimentation during regression and/or irregular sedimentary processes. Because of these uncertainties, an assumed phylogeny implies such gaps if two sister taxa have different times of first appearance or if a gap exists between the last appearance of an inferred ancestor and the first appearance of its inferred descendant [48]. Accordingly, we should not regard the first appearance of the first fossil record as the “timing” of the split, but rather that the split of two lineages was older than the age of the first fossil record (the younger limit). Additionally, in many cases, it is difficult to assume a particular split is younger than the confirmed age based on the fossil record (the older limit). For this reason, at first we estimated the divergence times of the goat and the markhor within the comprehensive evolutionary framework of the Cetartiodactyla using several reliable fossil records for calibration. The phylogenetic tree was inferred based on the amino acid sequences of the concatenated 12 mitochondrial protein coding genes encoded in the H strand with the RAxML v. 7.0.3 program using the mtREV+F+Г_4_ model [49]. Assuming the basal position of Camelidae [50], the divergence times were estimated using the relaxed clock model [51] using the MCMCTREE program [52] implemented in PAML v. 4.4. In this analysis, the normal approximation method was used to reduce the computational burden. To estimate the Hessian matrix (variance – covariance matrix of branch lengths), we used the mtmam+Г_5_ model [53] for the amino acid sequences using the CODEML program in PAML, and the GTR+Г_8_ model for the nucleotide sequence using the BASEML program in PAML, where the model was applied separately to each of the three codon positions. The independent rates model between ancestral and descendant lineages was also applied (e.g., [54]). The prior distributions were set as follows: For the amino acid sequence, rgene_gamma  =  (4, 5), and σ2_gamma  =  (1, 0.8). For the nucleotide sequence, rgene_gamma  =  (4, 7), and σ2_gamma  =  (1, 0.8).

The following species and reference sequences were included in this analysis Goat (*Capra hircus*: this study), markhor (*Capra falconeri*: this study), Gobi ibex (*Capra sibirica:* this study), sheep (*Ovis aries*: AY858379), chiru (*Pantholops hodgsonii*: NC_007441), cattle (*Bos taurus*: HQ184045), yak (*Bos grunniens*: GQ464260), Asian water buffalo (*Bubalus bubalis*: NC_006295), sika deer (*Cervus nippon centralis*: NC_006993), hippopotamus (*Hippopotamus amphibius*: AP003425), humpback whale (*Megaptera novaeangliae*:NC_006927), long beaked common dolphin (*Delphinus capensis*: NC_012061), pig (*Sus scrofa*: NC_012095), warthog (*Phacochoerus africanus*: NC_008830), collared peccary (*Pecari tajacu*: NC_012103), two-humped camel (*Camelus bactrianus*: NC_009628), lama (*Lama glama*: NC_012102), dog (*Canis familiaris:* EU789788), cat (*Felis catus*: FCU20753), and the horse (*Equus caballus:* EU939445). The fossil calibrations were as follows: The divergence between the Bovinae and Caprinae was from 18.3 to 28.5 Ma [20]. The divergence between Cetacea and the hippopotamus was from 52 to 58 Ma [55]–[57], the baleen whale and the toothed whale was older than 34.1 Ma [57]–[60]. Caniformia and Feliformia was between 42.8 and 63.8 Ma [20], Carnivora and Perissodactla was between 62.3 and 71.2 Ma [20]. The divergence of Cetartiodactyla and Carnivora+Perissodactla was younger than 113 Ma [20]. The fine-tune parameters were adjusted such that all acceptant rates were distributed from 0.2 to 0.4.

For the divergence time estimation among goats, the strict molecular clock method was applied, which assumes homogeneity of the substitution rate among lineages. Even if the evolutionary rate does not differ among lineages, evolutionary divergence cannot be read directly from the observed difference between two sequences because of multiple substitutions with extreme site heterogeneity [61]. In addition, as mentioned above, the substitution rates in intra-species comparisons are higher than that of inter-species comparisons. Ho et al. [62] demonstrated a high evolutionary rate in the short term (<1 Ma) and a low rate in the long term (>1 Ma), and approximated this rate change using the exponential decline curve. Williamson and Orive [63] investigated the impact of purifying selection on the shape of genealogy and the distribution of mutations. As a result they demonstrated that although the shapes of trees topology remained largely unchanged, the distributions of the mutations on the trees shifted. Since a majority of coalescent analyses assume neutrality, this effect of purifying selection could create a bias in associated estimations. Therefore, we used only 3^rd^ codon positions because most of the substitutions in the 3^rd^ codon positions are synonymous and are therefore likely to be neutral. Thus, they are thought to be relatively free from this rate change. The GTR+Г_4_ model was used for the present analysis. We confirmed that there are almost no multiple substitutions among the goat and markhor (data not shown) and estimated the split of the goat and markhor to be 3.4 Ma.

## Supporting Information

Figure S1 **The maximum likelihood tree of domestic goats based on the nearly complete mitochondrial protein-encoding genes of the smaller data set.** The GTR+I+Г_4_ model was used. Taking into account the different tempo and mode of nucleotide substitution, each of the three codon positions was analyzed separately. The branch lengths are proportional to numbers of nucleotide substitutions. The markhor was used as an outgroup. Nodal numbers indicate bootstrap probabilities (rapid bootstrap method: 1,000 replications).
(TIF)

Figure S2 **Divergence time estimates among Cetartiodactyla based on the amino acid sequences of the complete mitochondrial protein-encoding genes.** The nodal numbers indicate the estimated divergence times ± standard errors in Ma (mega-annum). Numbers in brackets indicate estimates based on nucleotide sequences. Calibrations are shown in angled brackets.
(TIF)

Figure S3 **Differences of ω ratios among goat lineages based on the larger data set of nearly complete mitochondrial protein-encoding genes.** The branch model analysis assuming different ω ratios in the shallow branches (a); and the branch model analysis assuming different ω ratios in the deep branches (b). The branch lengths are proportional to numbers of codon substitutions.
(PPT)

Table S1 **Tajima's D of each haplogroup based on the D-loop sequences of the wild bezoar.**
(XLS)

Table S2 **PCR primer pairs used for amplification of mitochondrial protein coding region and HV1 region.**
(XLS)

Table S3 **List of the variant sites.**
(XLSX)
