# Supplementary figures and images for: Domestication Process of the Goat Revealed by an Analysis of the Nearly Complete Mitochondrial Protein-Encoding Genes
Source: PLoS One. 2013 Aug 1;8(8):e67775. doi: 10.1371/journal.pone.0067775 (PMC3731342; doi:10.1371/journal.pone.0067775)

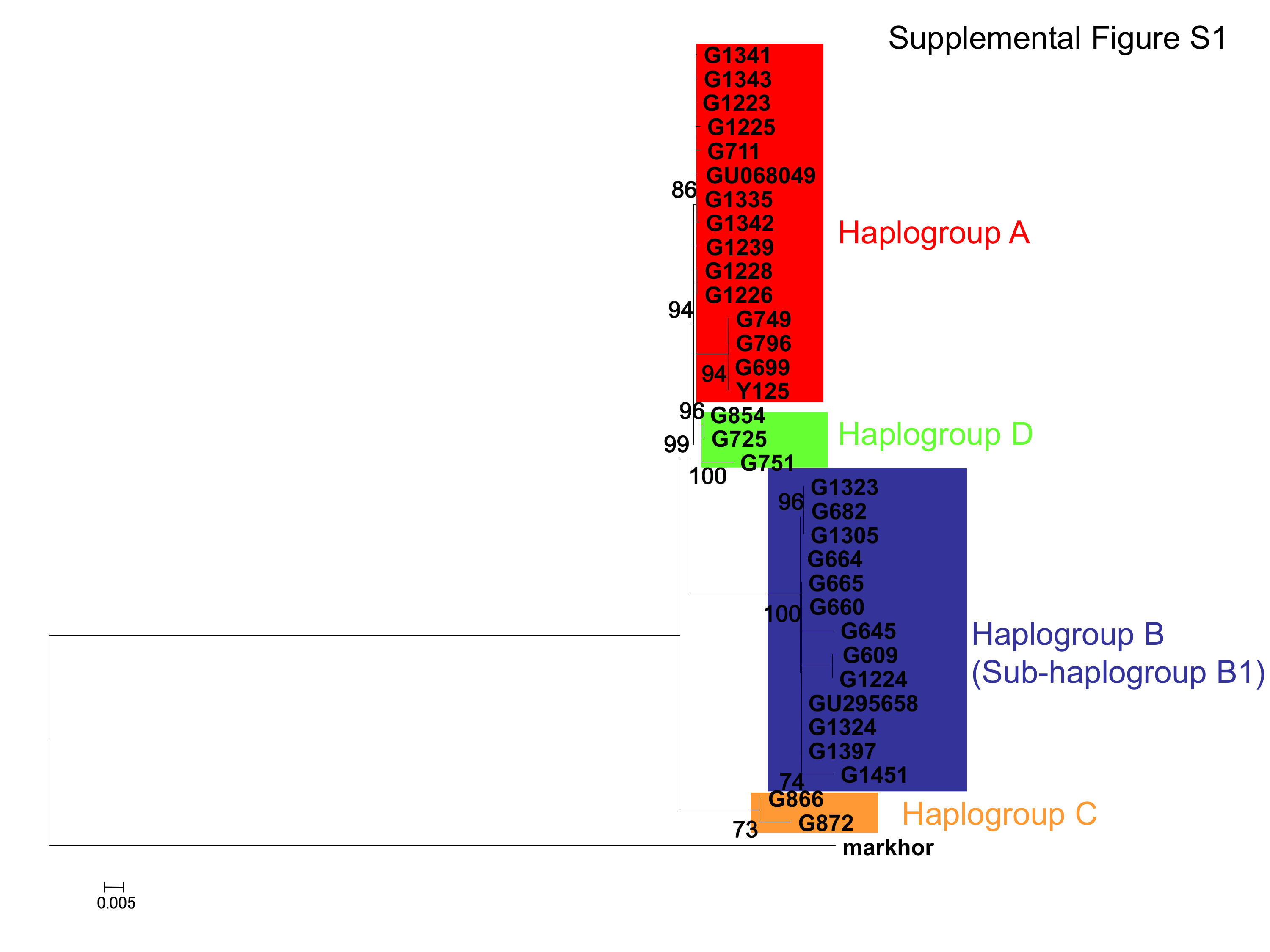

Supplement: Figure S1 — The maximum likelihood tree of domestic goats based on the nearly complete mitochondrial protein-encoding genes of the smaller data set. The GTR+I+Г4 model was used. Taking into account the different tempo and mode of nucleotide substitution, each of the three codon positions was analyzed separately. The branch lengths are proportional to numbers of nucleotide substitutions. The markhor was used as an outgroup. Nodal numbers indicate bootstrap probabilities (rapid bootstrap method: 1,000 replications). (TIF) [file pone.0067775.s001.tif]

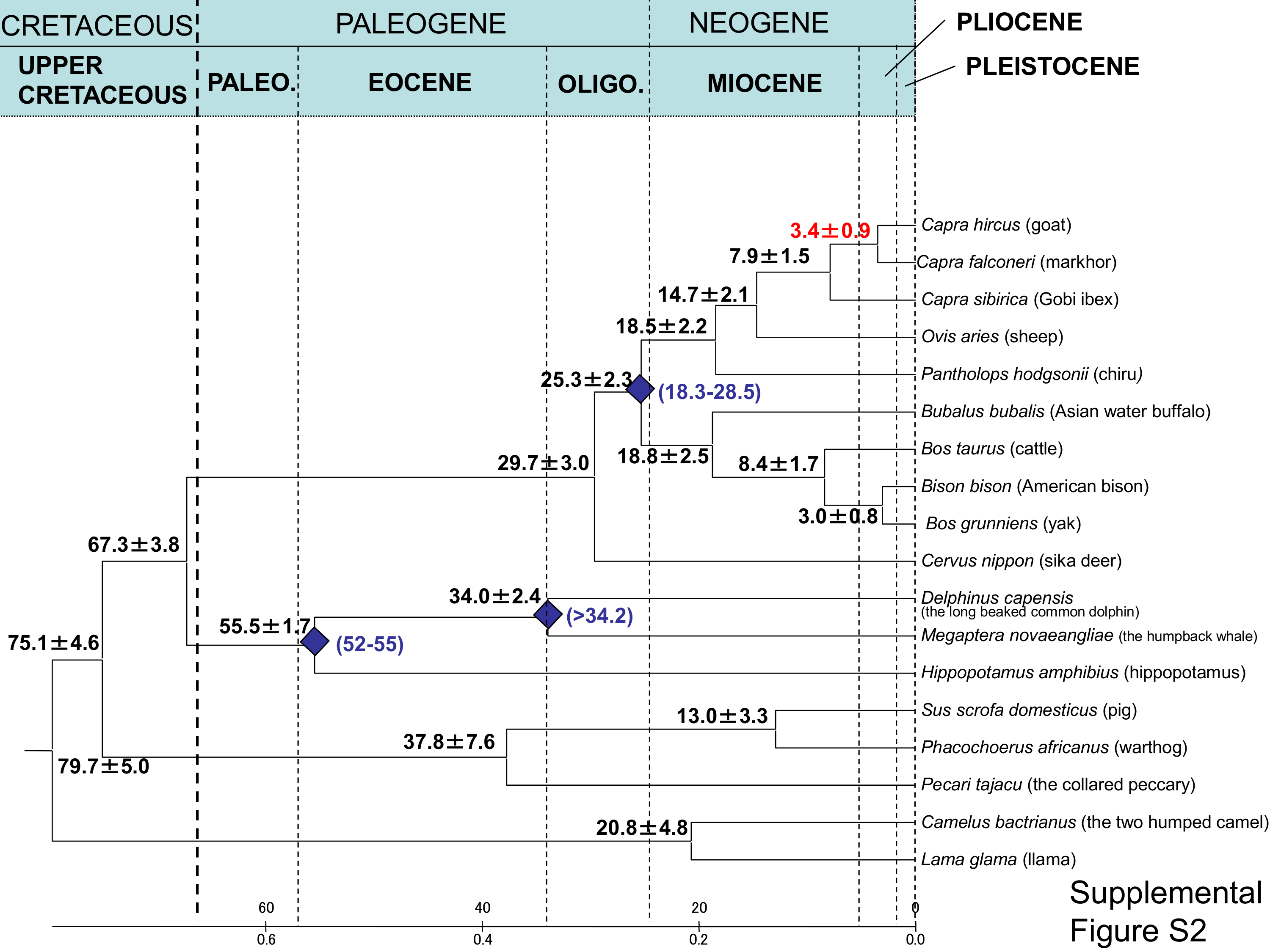

Supplement: Figure S2 — Divergence time estimates among Cetartiodactyla based on the amino acid sequences of the complete mitochondrial protein-encoding genes. The nodal numbers indicate the estimated divergence times ± standard errors in Ma (mega-annum). Numbers in brackets indicate estimates based on nucleotide sequences. Calibrations are shown in angled brackets. (TIF) [file pone.0067775.s002.tif]
