# Supplementary material for: Domestication Process of the Goat Revealed by an Analysis of the Nearly Complete Mitochondrial Protein-Encoding Genes
Source: PLoS One. 2013 Aug 1;8(8):e67775. doi: 10.1371/journal.pone.0067775 (PMC3731342; doi:10.1371/journal.pone.0067775)
Supplement: Figure S3 — Differences of ω ratios among goat lineages based on the larger data set of nearly complete mitochondrial protein-encoding genes. The branch model analysis assuming different ω ratios in the shallow branches (a); and the branch model analysis assuming different ω ratios in the deep branches (b). The branch lengths are proportional to numbers of codon substitutions. (PPT) [file pone.0067775.s003.ppt]

## Slide 1
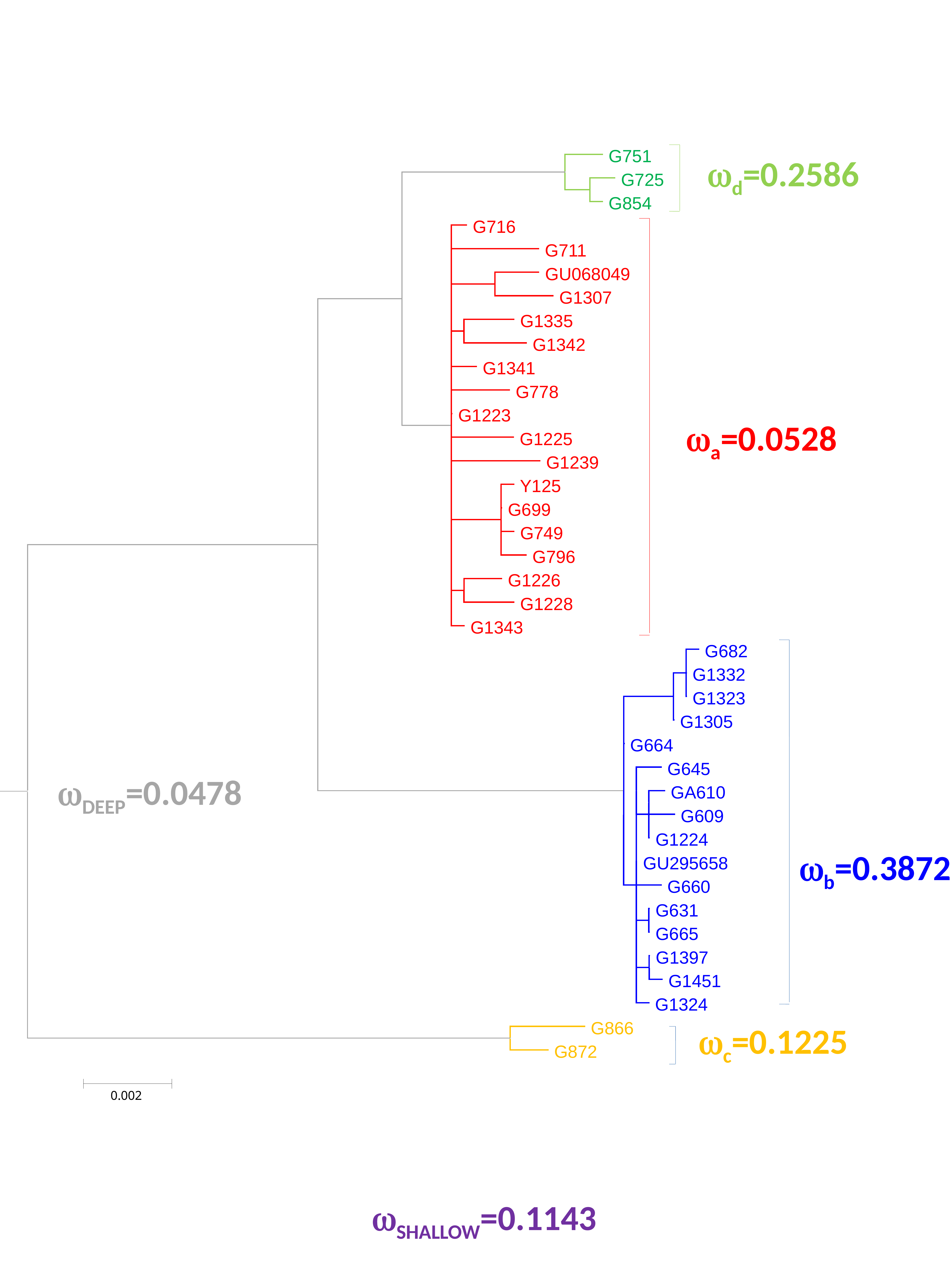

G751
 G725
 G854
 G716
 G711
 GU068049
 G1307
 G1335
 G1342
 G1341
 G778
 G1223
 G1225
 G1239
 Y125
 G699
 G749
 G796
 G1226
 G1228
 G1343
 G682
 G1332
 G1323
 G1305
 G664
 G645
 GA610
 G609
 G1224
 GU295658
 G660
 G631
 G665
 G1397
 G1451
 G1324
 G866
 G872
0.002
d=0.2586
a=0.0528
DEEP=0.0478
b=0.3872
c=0.1225
SHALLOW=0.1143

## Slide 2
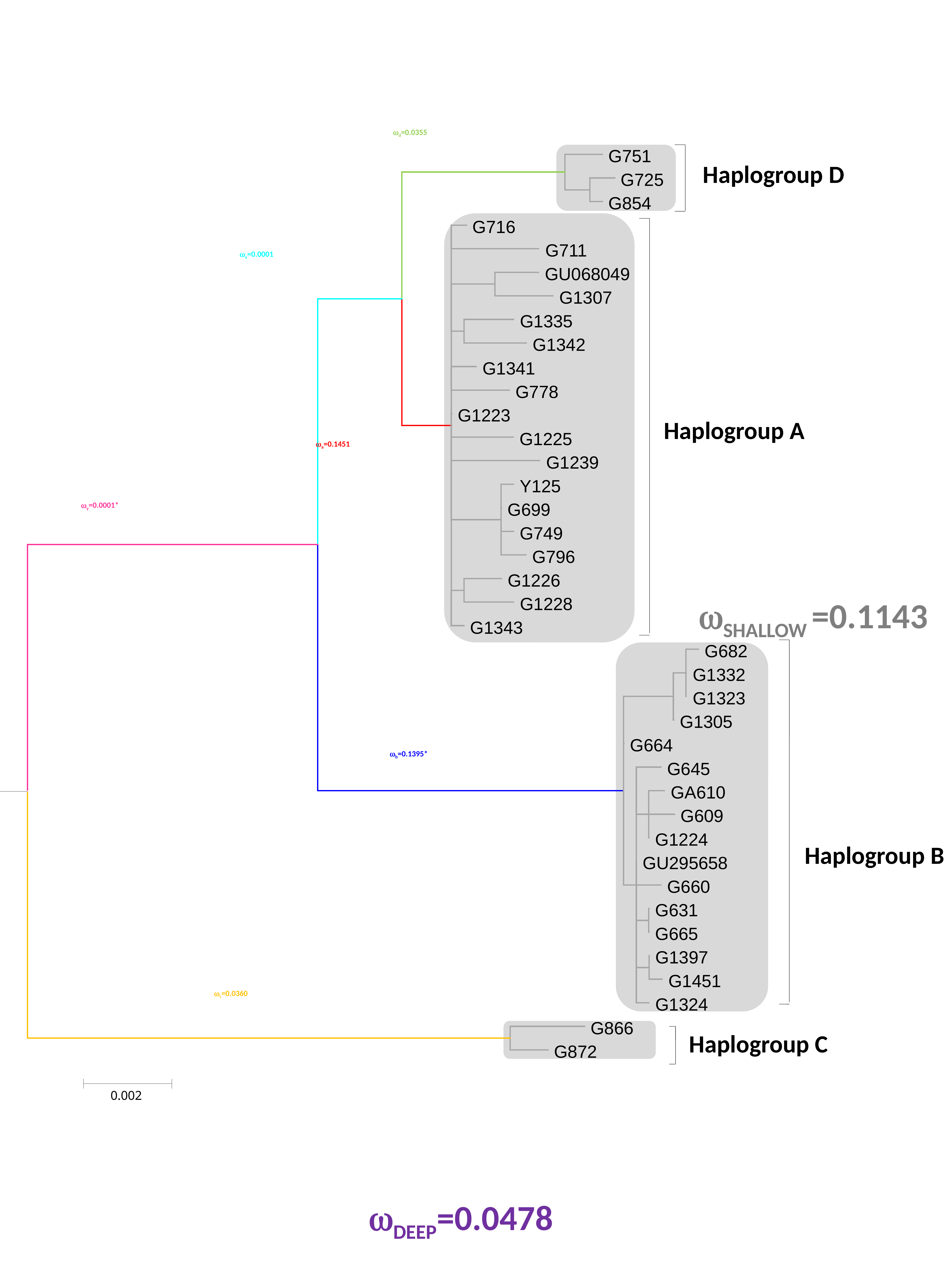

d=0.0355
 G751
Haplogroup D
 G725
 G854
 G716
 G711
 x=0.0001
 GU068049
 G1307
 G1335
 G1342
 G1341
 G778
 G1223
Haplogroup A
 G1225
 a=0.1451
 G1239
 Y125
 y=0.0001*
 G699
 G749
 G796
 G1226
SHALLOW =0.1143
 G1228
 G1343
 G682
 G1332
 G1323
 G1305
 G664
 b=0.1395*
 G645
 GA610
 G609
 G1224
Haplogroup B
 GU295658
 G660
 G631
 G665
 G1397
 G1451
 c=0.0360
 G1324
 G866
Haplogroup C
 G872
0.002
DEEP=0.0478
